# Supplementary material for: Unravelling the amorphous structure and crystallization mechanism of GeTe phase change memory materials
Source: Nat Commun. 2024 Feb 3;15:1011. doi: 10.1038/s41467-024-45327-7 (PMC10837456; doi:10.1038/s41467-024-45327-7)
Supplement: Supplementary file 1 — Supplementary Information [file 41467_2024_45327_MOESM1_ESM.pdf]

Supplementary Information

Unravelling the Amorphous Structure and Crystallization Mechanism of GeTe Phase Change Memory Materials

Simon Wintersteller[1], Olesya Yarema[2], Dhananjeya Kumaar[1], Florian M. Schenk[1], Olga V. Safonova[3], Paula M. Abdala[4], Vanessa Wood[2], and Maksym Yarema[1,*]

[1] Chemistry and Materials Design, Institute for Electronics, Department of Information Technology and Electrical Engineering, ETH Zürich, 8092 Zürich, Switzerland

[2] Materials and Device Engineering, Institute for Electronics, Department of Information Technology and Electrical Engineering, ETH Zürich, 8092 Zürich, Switzerland

[3] Paul Scherrer Institute, 5232 Villigen, Switzerland

[4] Laboratory of Energy Science and Engineering, Department of Mechanical and Process Engineering, ETH Zurich, 8092 Zurich, Switzerland

* Correspondence to yaremam@ethz.ch

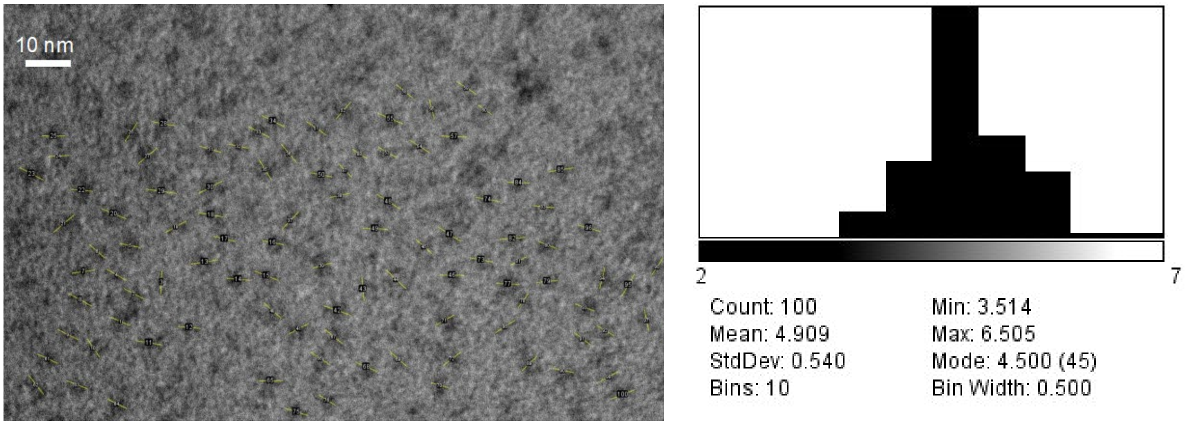

Supplementary Figure 1 | Size quantification of ZnS-coated GeTe nanoparticles from transmission electron microscopy (TEM) images. The nanoparticles have a mean diameter of 4.9 nm with a standard deviation of 0.54 nm.

Supplementary Table 1 | EDX quantification of GeTe microparticles and nanoparticles. Both cases demonstrate good 1:1 stoichiometry of Ge and Te atoms as well as a low concentration of Zn and S atoms for the nanoparticles, indicating a thin* ZnS surface shell.

	GeTe microparticles		ZnS-coated GeTe nanoparticles	
**Element**	**Composition (at. %)**	**Error (%)**	**Composition (at. %)**	**Error (%)**
Ge	51.35	1.14	45.97	1.74
Te	48.65	2.11	47.17	1.23
Zn	–	–	5.38	5.59
S	–	–	1.47	31.35

* – From EDX measurements, we note that the ZnS shell corresponds to approximately 7 at. % of the nanoparticle. From this, we can estimate the thickness of the ZnS shell, using the formula $R_{NP}^3 = (1 - t_{shell})R_{tot}^3$, where R_{NP} and R_{tot} are radii of GeTe core and a total radius of nanoparticle and t_{shell} is a shell thickness, estimated here to approx. 1Å (i.e., sub-monolayer).

(a) GeTe microparticles

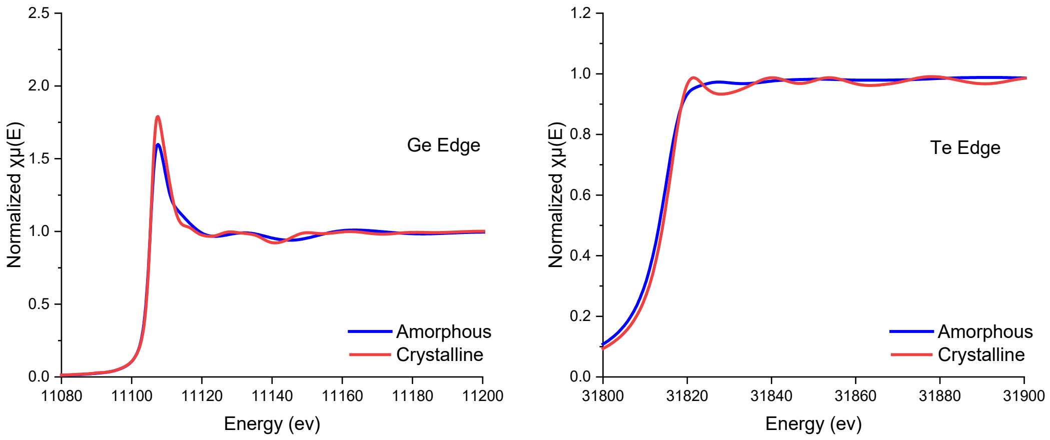

(b) GeTe nanoparticles

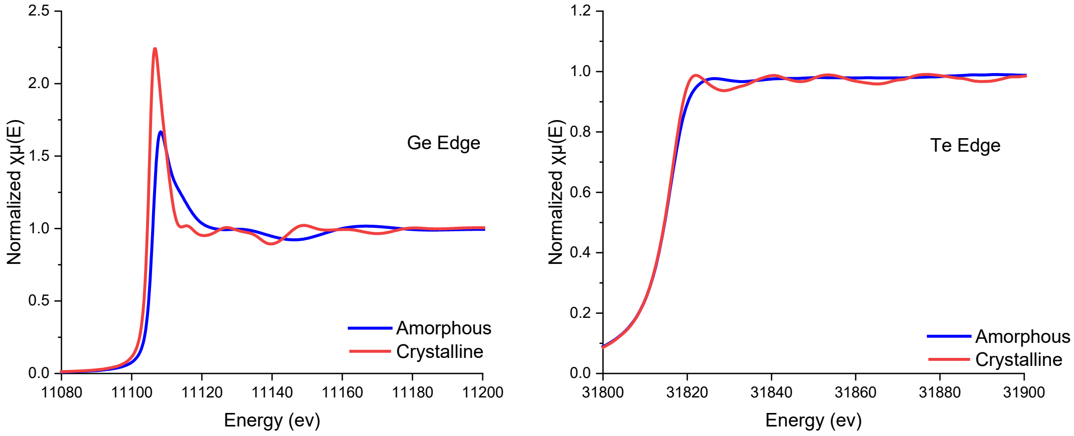

Supplementary Figure 2 | Normalized XAS spectra of amorphous and crystalline GeTe, measured at room temperature: (a) GeTe microparticles and (b) GeTe nanoparticles. Data are presented for both the Ge and Te K-edges.

(a) Amorphous GeTe microparticles

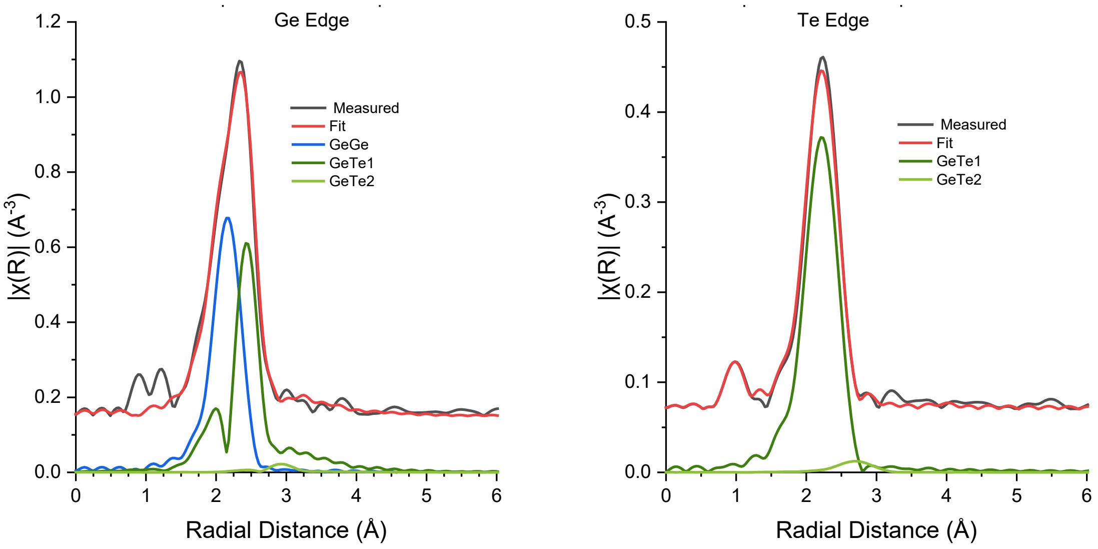

(b) Crystalline GeTe microparticles

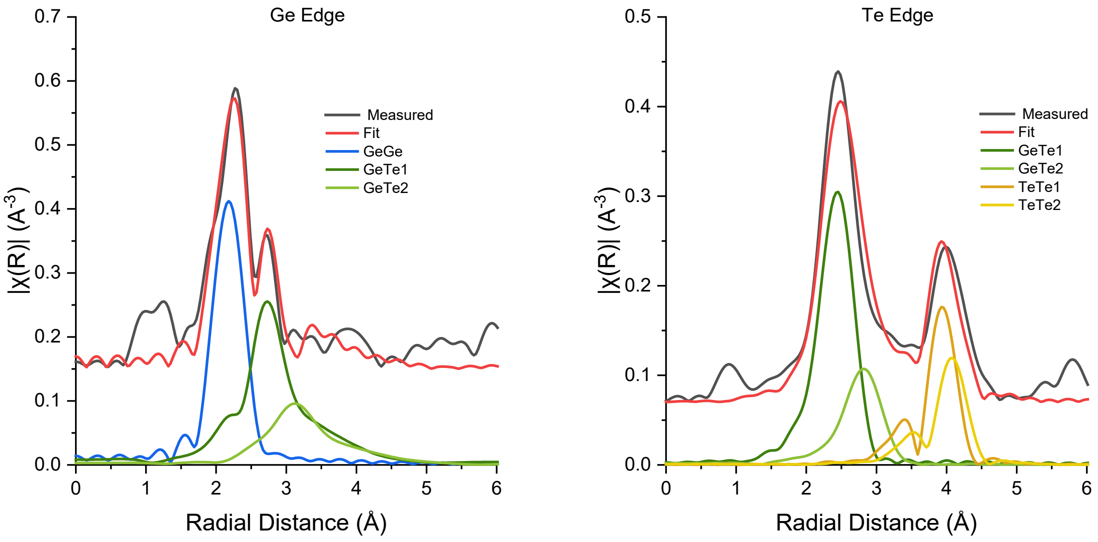

Supplementary Figure 3 | Fittings of XAS spectra, measured at room temperature for (a) amorphous and (b) crystalline GeTe microparticles for both the Ge and Te K-edge. In total 5 scattering paths are fitted: homopolar GeGe bond, short and elongated GeTe bond, and in addition the second nearest neighbors for the crystalline Te edge (two TeTe bonds).

(a) Amorphous GeTe nanoparticles

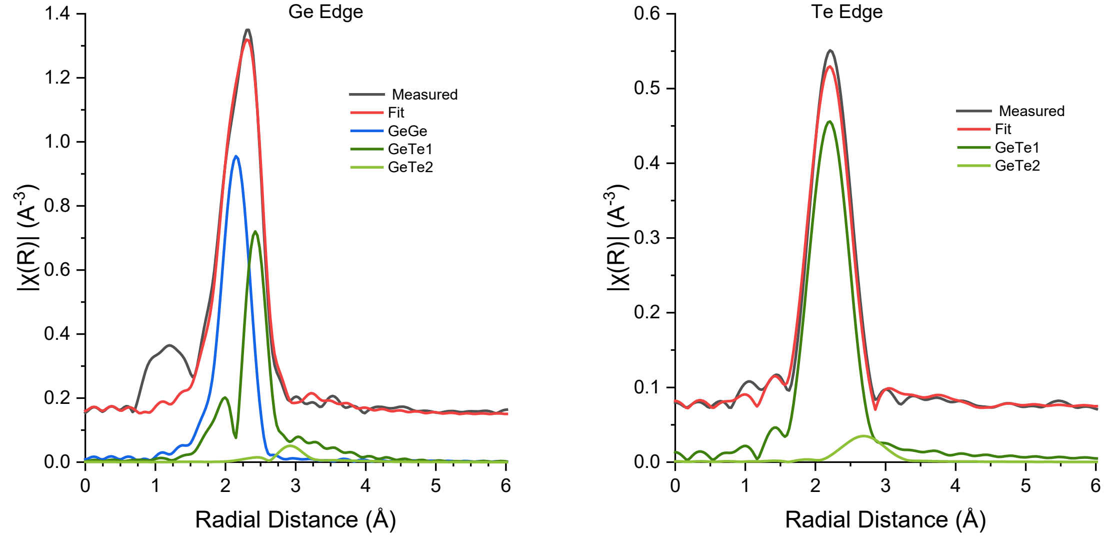

(b) Crystalline GeTe nanoparticles

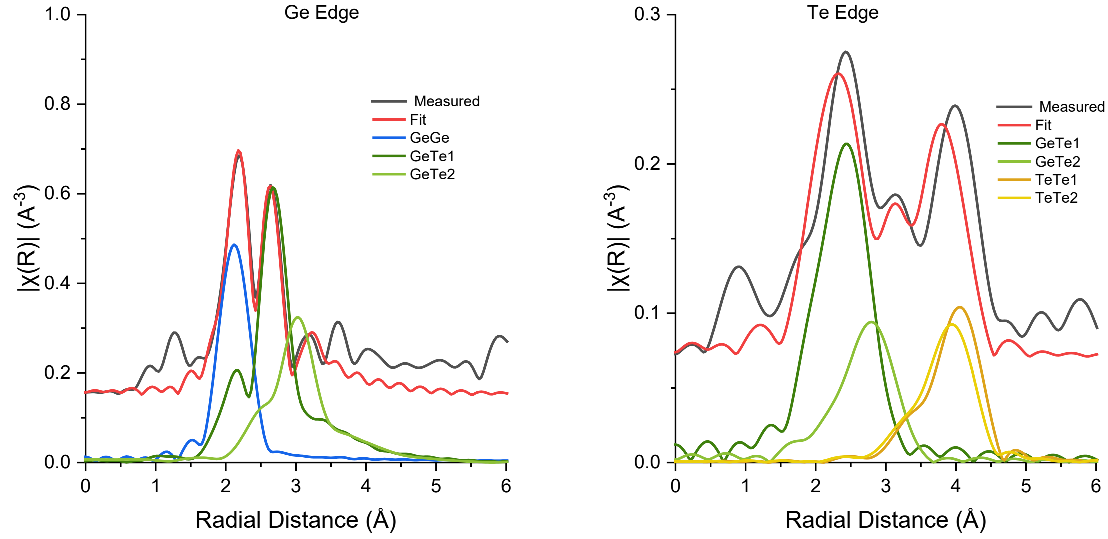

Supplementary Figure 4 | Fittings of XAS spectra, measured at room temperature for (a) amorphous and (b) crystalline GeTe nanoparticles for both the Ge and Te K-edge. In total 5 scattering paths are fitted: homopolar GeGe bond, short and elongated GeTe bond, and in addition the second nearest neighbors for the crystalline Te edge (two TeTe bonds).

Supplementary Table 2 | EXAFS fitting results of crystalline GeTe microparticles, measured at room temperature and for the Ge and Te K-edge.

		Crystalline GeTe microparticles							
**Edge**	**Scattering path**	**CN**		**σ^2 (10^{-3} Å^{-2})**		**Δe_0 (eV)**		**R (Å)**	
Ge edge	Ge-Ge	0.53	±0.05	0.70	-	7.51	±1.04	2.57	±0.01
	Ge-Te1	2.03	±0.40	17.29	±3.09	7.51	±1.04	2.83	±0.01
	Ge-Te2	1.82	±0.71	27.34	±4.88	7.51	±1.04	3.21	±0.02
Te edge	Ge-Te1	2.03	±0.40	7.54	±1.50	3.09	±1.39	2.83	±0.01
	Ge-Te2	1.82	±0.71	11.92	±2.36	3.09	±1.39	3.21	±0.02
	Te-Te1	5.40	±1.37	10.95	±2.17	3.09	±1.39	4.18	±0.02
	Te-Te2	5.40	±1.37	13.38	±2.65	3.09	±1.39	4.32	±0.04
R factor		0.0391							

Supplementary Table 3 | EXAFS fitting results of crystalline GeTe nanoparticles, measured at room temperature and for the Ge and Te K-edge.

		Crystalline GeTe nanoparticles							
**Edge**	**Scattering path**	**CN**		**σ^2 (10^{-3} Å^{-2})**		**Δe_0 (eV)**		**R (Å)**	
Ge edge	Ge-Ge	0.61	±0.05	0.70	-	3.00	±1.01	2.53	±0.01
	Ge-Te1	3.10	±0.53	12.15	±1.65	3.00	±1.01	2.79	±0.01
	Ge-Te2	2.55	±0.94	19.20	±2.61	3.00	±1.01	3.14	±0.02
Te edge	Ge-Te1	3.10	±0.53	11.54	±4.85	-0.60	±4.30	2.79	±0.01
	Ge-Te2	2.55	±0.94	18.23	±7.66	-0.60	±4.30	3.14	±0.02
	Te-Te1	8.09	±9.53	16.75	±7.04	-0.60	±4.30	4.23	±0.15
	Te-Te2	8.09	±9.53	20.47	±8.60	-0.60	±4.30	4.09	±0.17
R factor		0.0754							

Supplementary Table 4 | EXAFS fitting results of amorphous GeTe microparticles, measured at room temperature and for the Ge and Te K-edge.

		Amorphous GeTe microparticles							
**Edge**	**Scattering path**	**CN**		**σ^2 (10^{-3} Å^{-2})**		**Δe_0 (eV)**		**R (Å)**	
Ge edge	Ge-Ge	1.27	±0.06	3.74	-	2.49	±0.59	2.36	±0.00
	Ge-Te1	1.47	±0.10	5.89	±0.64	2.49	±0.59	2.62	±0.00
	Ge-Te2	0.12	±0.11	9.31	±1.00	2.49	±0.59	3.11	±0.06
Te edge	Ge-Te1	1.47	±0.10	5.30	±0.55	-0.15	±1.03	2.62	±0.00
	Ge-Te2	0.12	±0.11	8.38	±0.87	-0.15	±1.03	3.11	±0.06
	Te-Te1	0.09	±0.50	7.69	±0.80	-0.15	±1.03	4.22	±0.28
	Te-Te2	0.09	±0.50	9.40	±0.98	-0.15	±1.03	4.47	±0.47
R factor		0.0074							

Supplementary Table 5 | EXAFS fitting results of amorphous GeTe nanoparticles, measured at room temperature and for the Ge and Te K-edge.

		Amorphous GeTe nanoparticles							
**Edge**	**Scattering path**	**CN**		**σ^2 (10^{-3} Å^{-2})**		**Δe_0 (eV)**		**R (Å)**	
Ge edge	Ge-Ge	1.75	±0.10	3.74	-	3.16	±0.85	2.46	±0.00
	Ge-Te1	1.66	±0.17	5.74	±0.88	3.16	±0.85	2.60	±0.01
	Ge-Te2	0.27	±0.18	9.07	±1.40	3.16	±0.85	3.10	±0.05
Te edge	Ge-Te1	1.66	±0.17	3.30	±1.05	1.82	±1.62	2.60	±0.01
	Ge-Te2	0.27	±0.18	5.22	±1.66	1.82	±1.62	3.10	±0.05
	Te-Te1	0.20	±1.05	4.79	±1.53	1.82	±1.62	4.30	±0.33
	Te-Te2	0.20	±1.05	5.86	±1.87	1.82	±1.62	4.59	±0.50
R factor		0.0118							

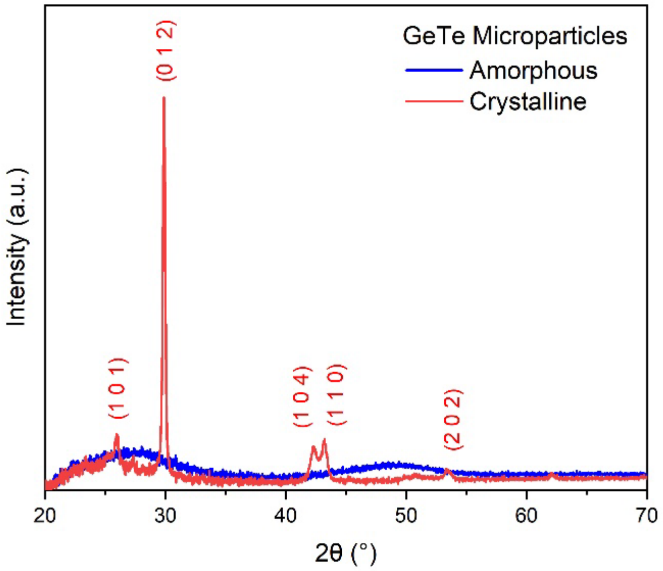

Supplementary Figure 5 | X-ray diffractogram of amorphous and crystalline GeTe microparticles. At room temperature, the crystalline GeTe is a rhombohedral phase with the space group R3mH.

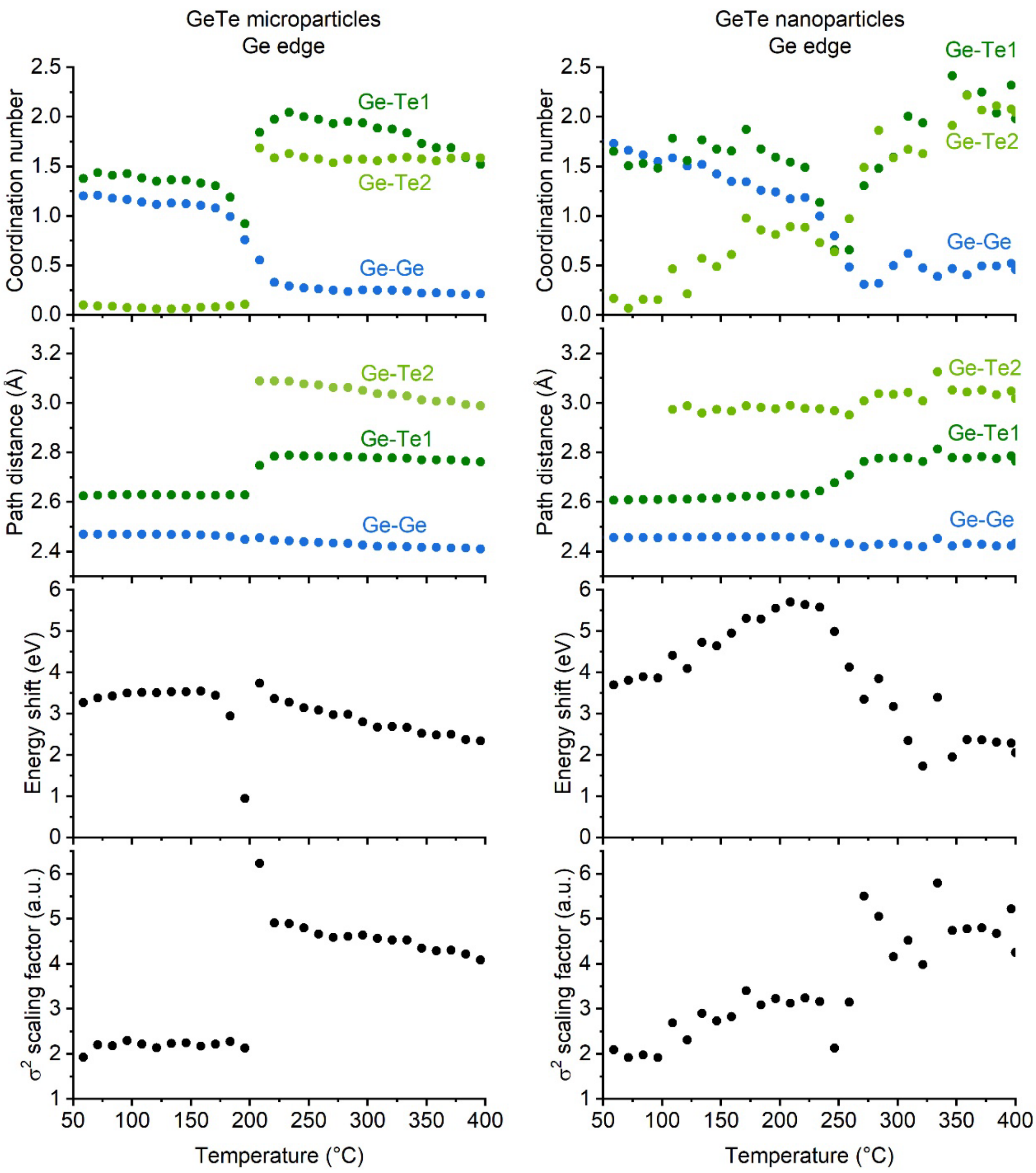

Supplementary Figure 6 | EXAFS fittings for GeTe microparticles and nanoparticles, while heating with the constant 5 °C/min ramp. Fitting results include the global coordination numbers and path distances as well as edge dependent energy shift and scaling factor of the mean square relative displacements (MSRDs). Ramp measurements were performed only on the Ge K-edge and hence only the GeGe, GeTe1 and GeTe2 paths can be fitted.

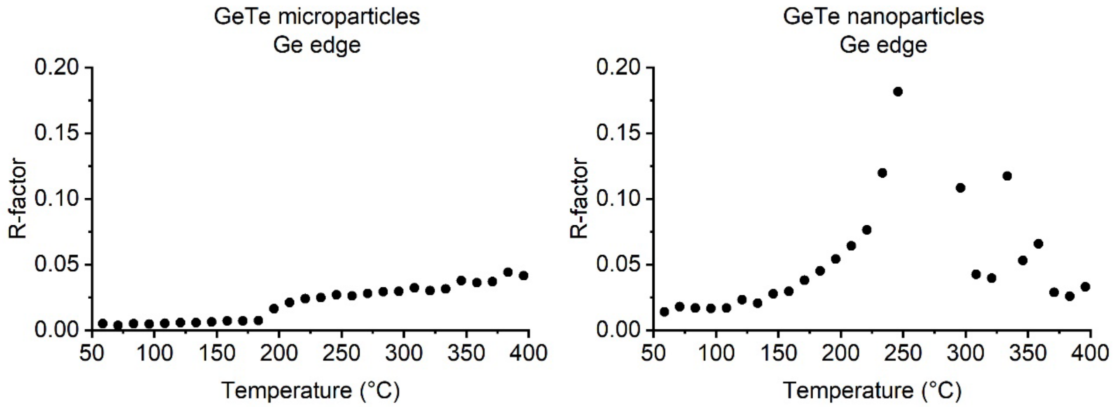

Supplementary Figure 7 | R-factors for the EXAFS fittings for GeTe microparticles and nanoparticles, presented in Supplementary Figure 6.

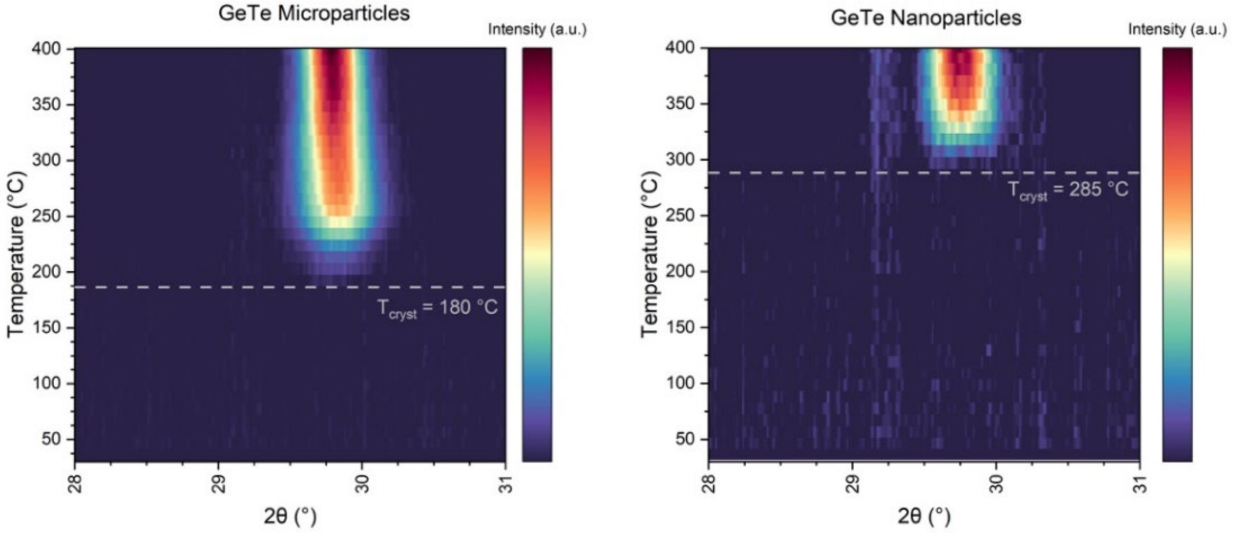

Supplementary Figure 8 | High-temperature X-ray diffraction (HTXRD) measurement of amorphous GeTe microparticles and nanoparticles, while heating with the constant 5 °C/min ramp. The HTXRD measurements determine crystallization temperature as the intensity onset of the main crystalline (202) diffraction peak. In a good agreement with XAS data, we observe notably higher crystallization temperature for GeTe nanoparticles. Small temperature offsets between XRD and XAS can be explained by a thermal lag during the XAS ramp measurements as well as different heating set-ups for the two methods. Note that for both GeTe bulk and GeTe nano, we observed the same distorted rock salt crystal structure, implying that ZnS shell does not affect the structure of the GeTe core of nanoparticles.

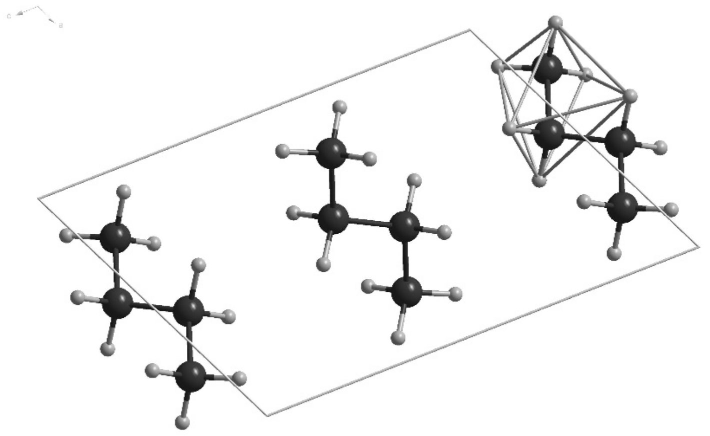

Supplementary Figure 9 | Unit cell of butane C_4H_{10} with highlighted distorted octahedral cage, made of H atoms. C atoms in black, H atoms in gray.

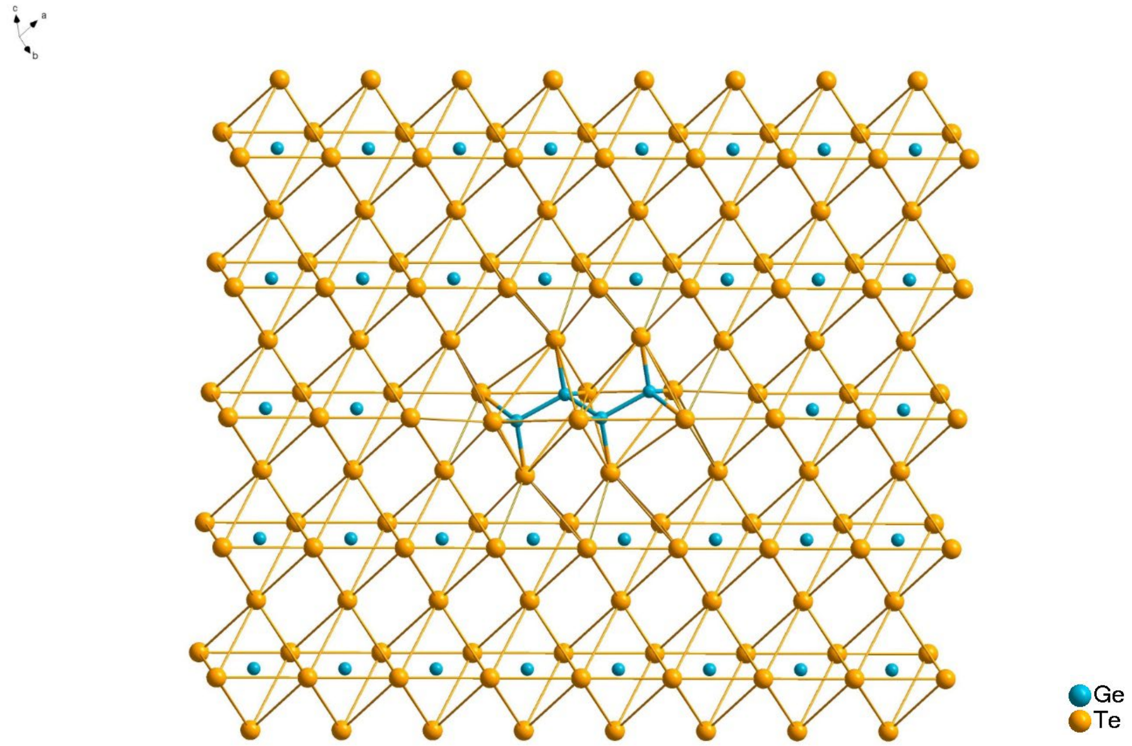

Supplementary Figure 10 | Butane-like Ge_4Te_{10} structural unit within the distorted rock-salt GeTe structure. Note two empty Te_6 octahedra next to two Te_6 disordered octahedra hosting 2 Ge atoms. Importantly, the tunneling of Ge atom to the neighboring octahedron is associated with relatively small distortions of very few Te atoms.

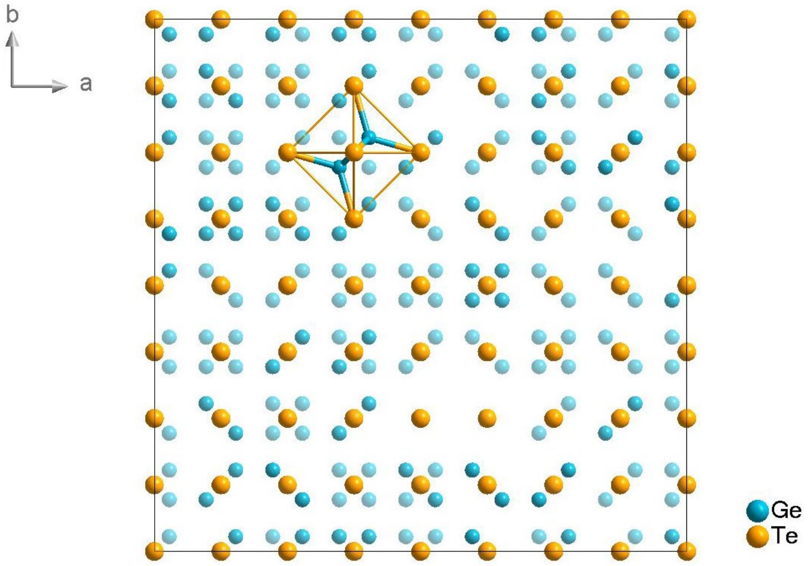

Supplementary Figure 11 | The 512-atom starting structure for the amorphous GeTe modelling with 25.3244 Å lattice vectors, comprising a 4×4×4 net of Te octahedra. Half of Te_6 octahedra is empty, while the other half is populated with 2 Ge atoms, forming prototypical Ge_2Te_6 ethane-like units (highlighted).

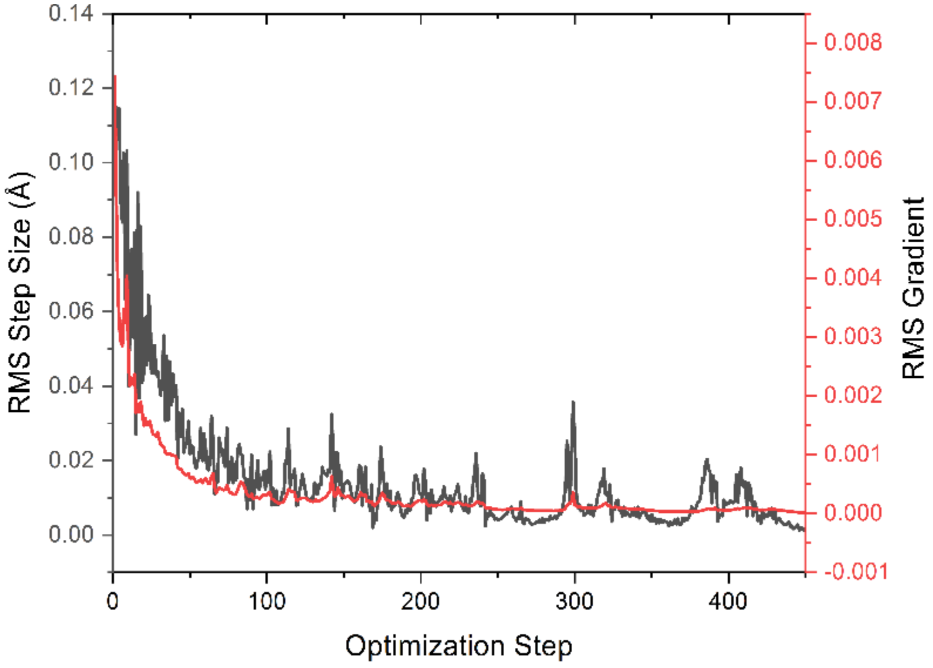

Supplementary Figure 12 | Geometry optimization of the 512-atom model with 0.031524 atom/$Å^3$ atomic density and PBC. During geometry optimization the structure converges after 464 steps reaching the CP2K default convergence values of 0.00045 RMS step size and 0.0003 RMS gradient.

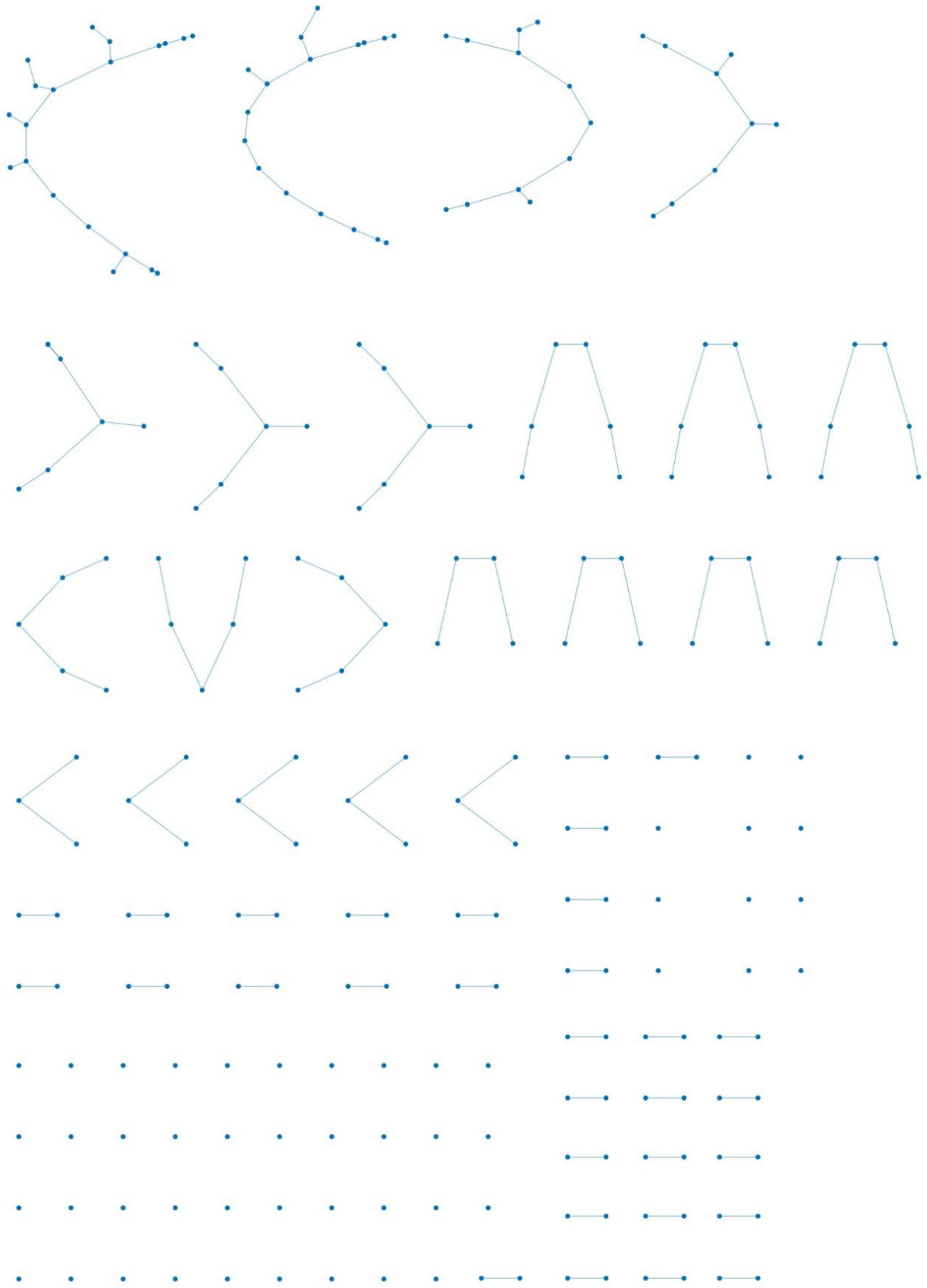

Supplementary Figure 13 | Distribution of Ge chains in one of 512-atom GeTe amorphous structures. Bond length and branching are reminiscent of organic chains (distances between Ge atoms are not to scale).

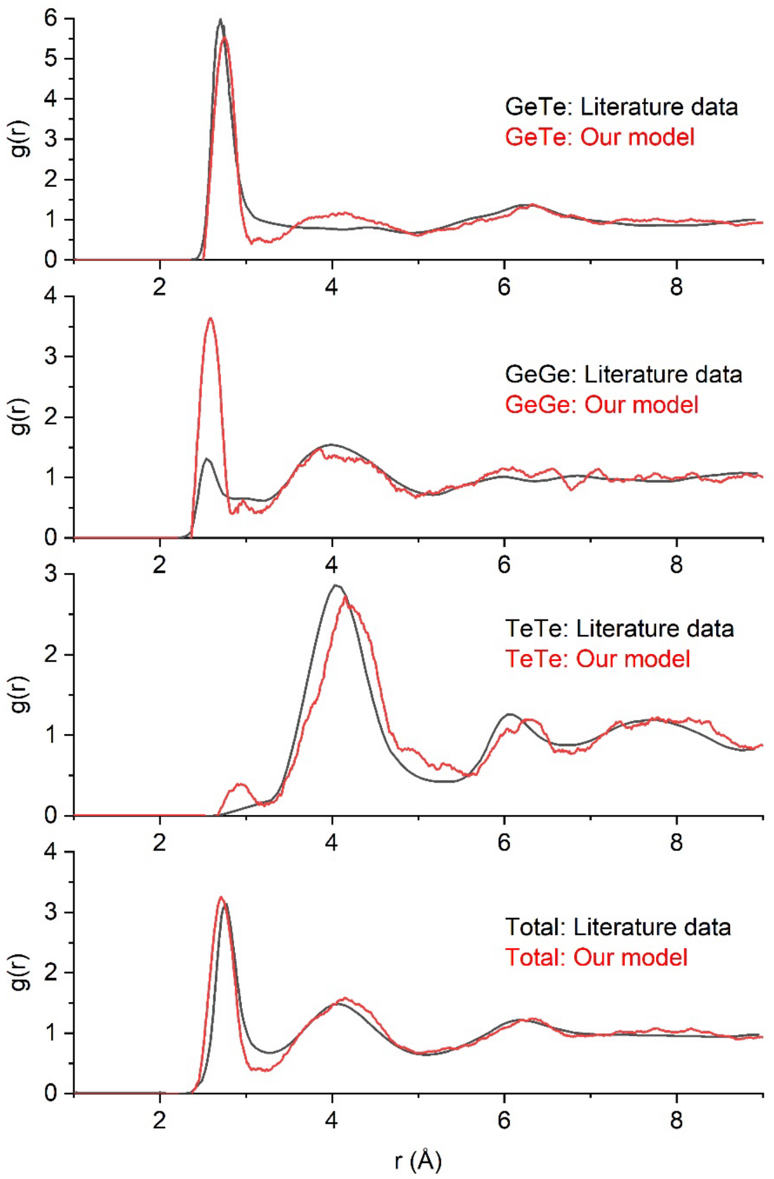

Supplementary Figure 14 | Total and partial pair correlation functions of the amorphous model in comparison to literature.[1] We observe a stronger interaction of Ge-Ge bonds as well as incrementally better-defined bond distances in comparison to the AIMD literature model.

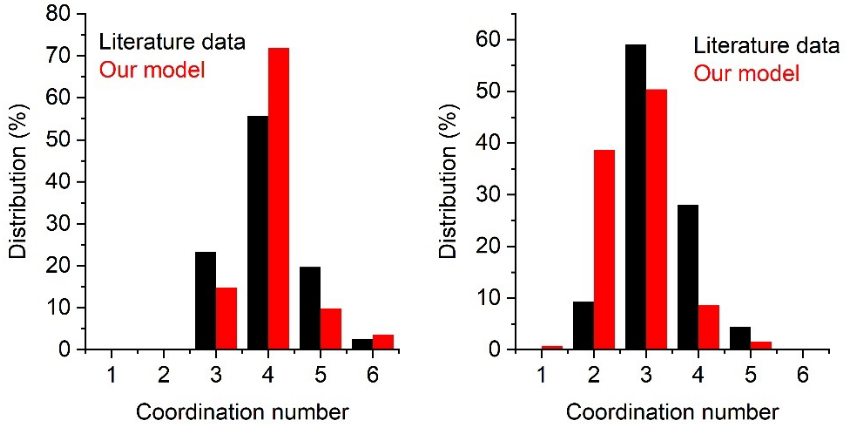

Supplementary Figure 15 | Coordination environments for Ge and Te atoms, derived from our models and from the literature data.[1] For direct comparison, we adopted the literature-defined bond cut-off distances of 3.0, 3.0 and 3.22 Å, for the Ge-Ge, Te-Te and Ge-Te bonds respectively. We observe very similar coordination environments for Ge atoms with the major difference being the larger concentration of 2-fold coordinated Te atoms corresponding to Ge-Te-Ge bridge states.

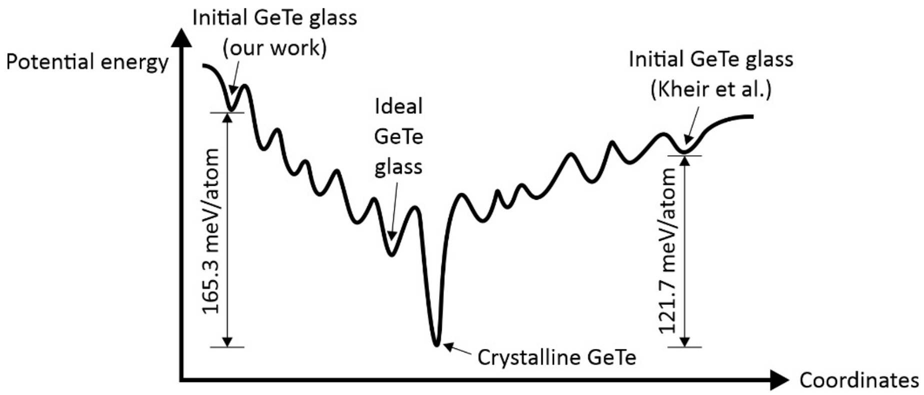

Supplementary Figure 16 | Energetics of the amorphous GeTe, comparing our results and the literature.[2] Our DFT calculations reveal –164.6498 and –164.4845 eV/atom for the crystalline and amorphous structure respectively, corresponding to an energy difference of 165.3 meV/atom. An energy difference per atom of 121.7 meV for the crystalline and amorphous phase is determined from AIMD melt-quench model. 095004). This suggests that our initial GeTe structure is characteristic of a higher degree of amorphousness.

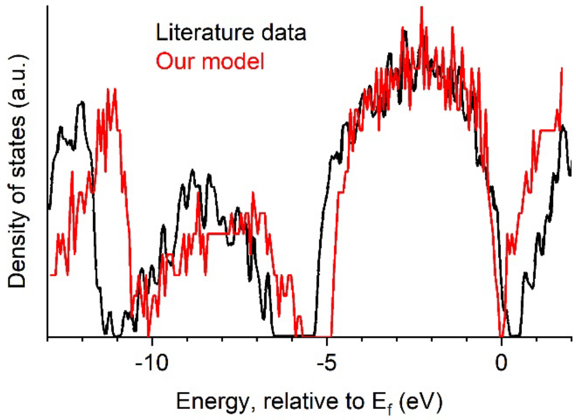

Supplementary Figure 17 | Electronic density of states (DOS) of our amorphous model in comparison to AIMD literature structure.[1] We observe a smaller bandgap due to the higher concentration of tetrahedral Ge and Ge-Te-Ge bridge defect states in our model.

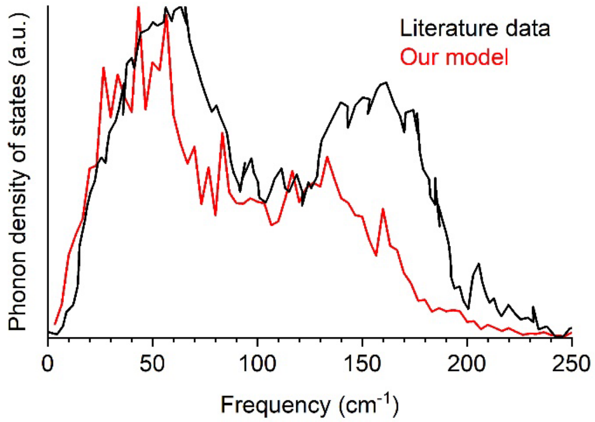

Supplementary Figure 18 | Phonon density of states (PDOS) of our amorphous model in comparison to AIMD literature structure. [1] We observe fewer high-frequency contributions around 150 cm^{-1} corresponding to the concentration of octahedral Ge environments.

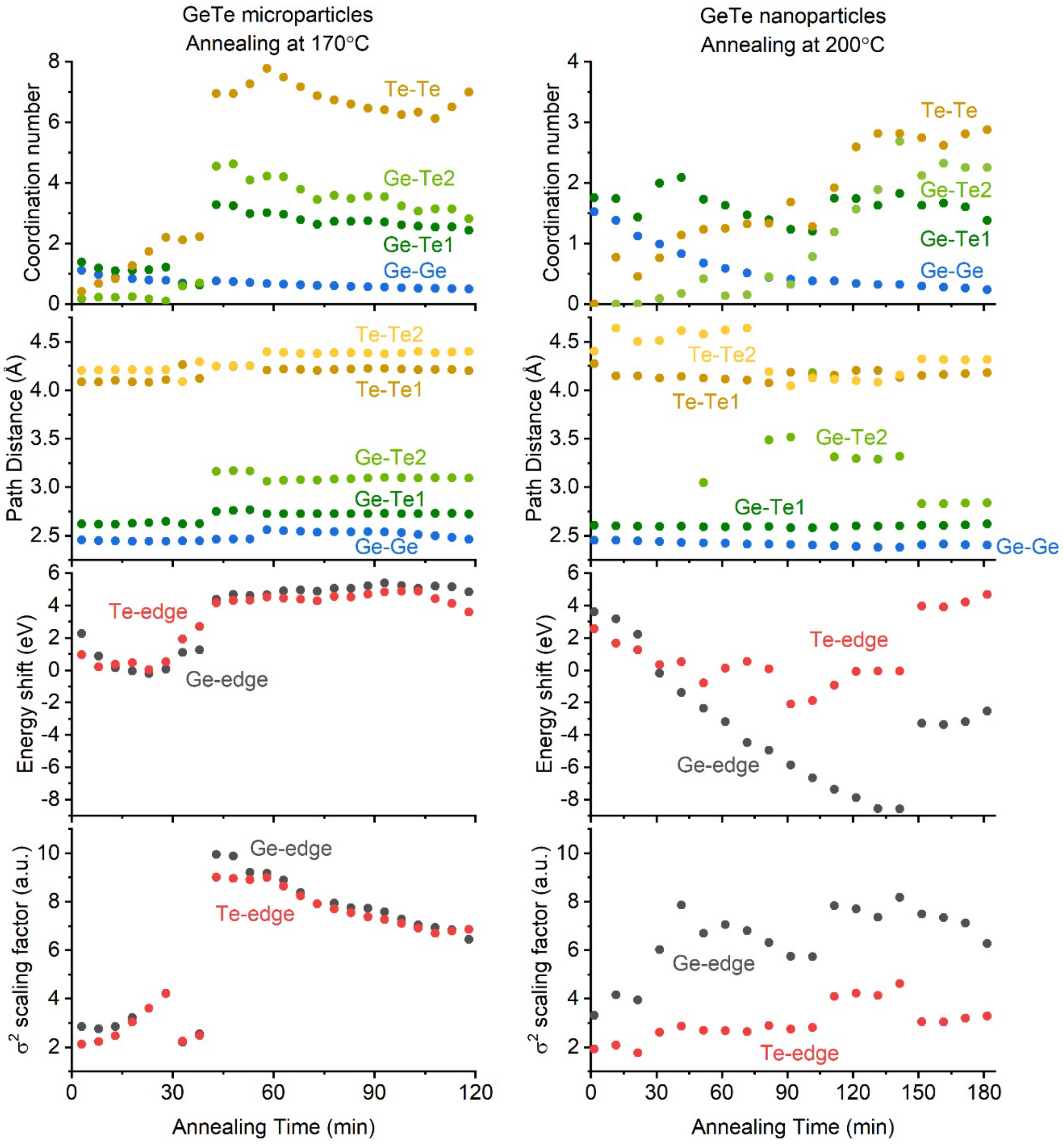

Supplementary Figure 19 | EXAFS fittings for GeTe microparticles and nanoparticles, while annealing at 170 and 200°C, respectively. Fitting results include the global coordination numbers and path distances as well as edge dependent energy shift and scaling factors of the mean square relative displacements (MSRDs). For the annealing measurements, both Ge and Te K-edges are measured allowing the fitting of the 2nd nearest neighbors for the Te atoms (TeTe1 and TeTe2). Furthermore, multi-edge global fittings enable the coupling of path parameters, such as coordination number and path distance for the GeTe1 and GeTe2 bonds for Ge and Te K-edge, enabling more accurate fits.

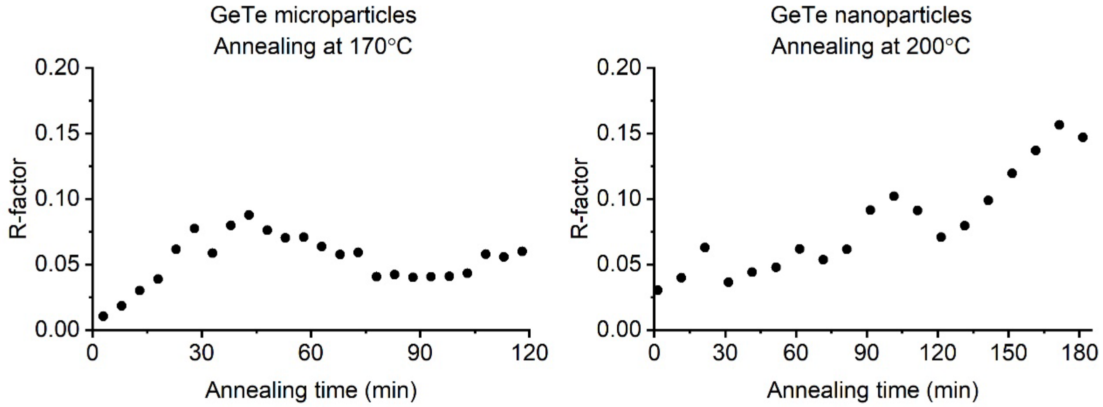

Supplementary Figure 20 | R-factor for the EXAFS fittings for GeTe microparticles and nanoparticles, presented in Supplementary Figure 19.

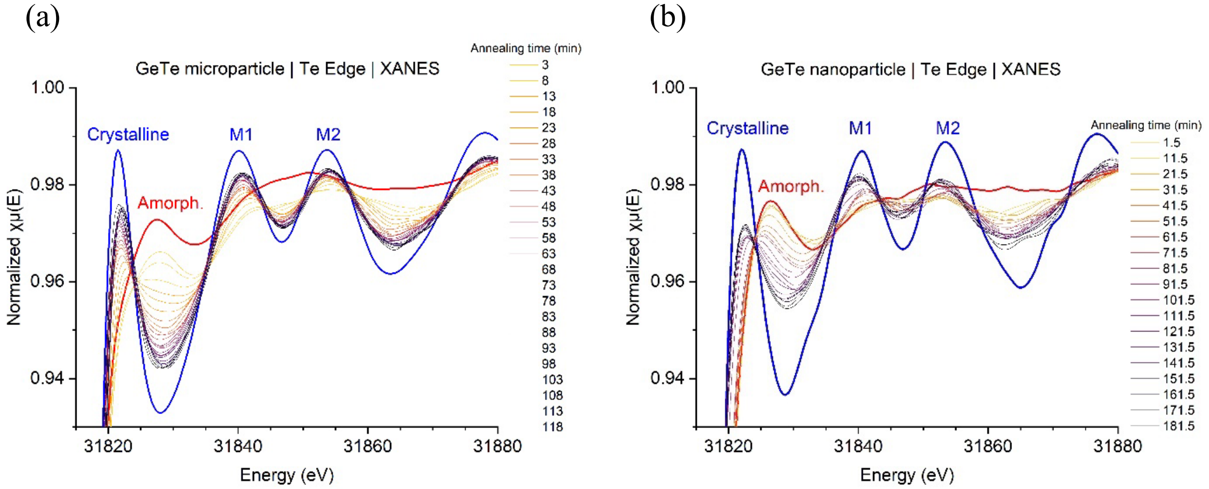

Supplementary Figure 21 | Normalized in-situ annealing XANES spectra of (a) GeTe microparticles at 170 °C and (b) GeTe nanoparticles at 200 °C. We present the Te K-edge here to observe a temporal evolution of peaks around 31840 and 31855 eV corresponding to the ordering of the *fcc*-type Te sublattice, prior to crystallization. Designation M1 nd M2 of *fcc*-type lattice are taken form literature.[3]

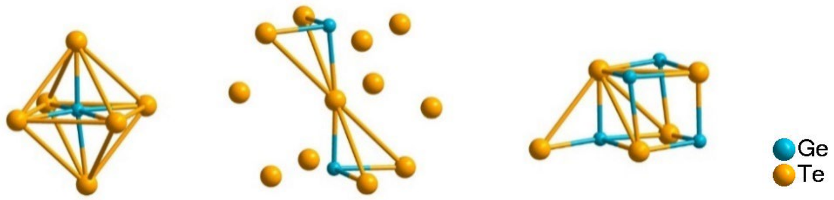

Supplementary Figure 22 | Hypothetic structural units of the GeTe ideal glass, where 1/3 of Te atoms attain the ordering of crystalline GeTe phase (i.e., 4-out-of-12 Te-Te bonds resemble crystalline GeTe). There are many ways in which 4-out-of-12 Te-Te bonds may order. For example, through an array of multiple supercritical nucleation centers, such as unlinked Te octahedra (left panel of Supplementary Figure 22) or Ge_4Te_4 cuboids, which are geometrically correlated via Ge-Te-Ge bridges (right panel of Supplementary Figure 22). Central panel of Supplementary Figure 22 shows an example of 2-dimensional ordering of Te sublattice.

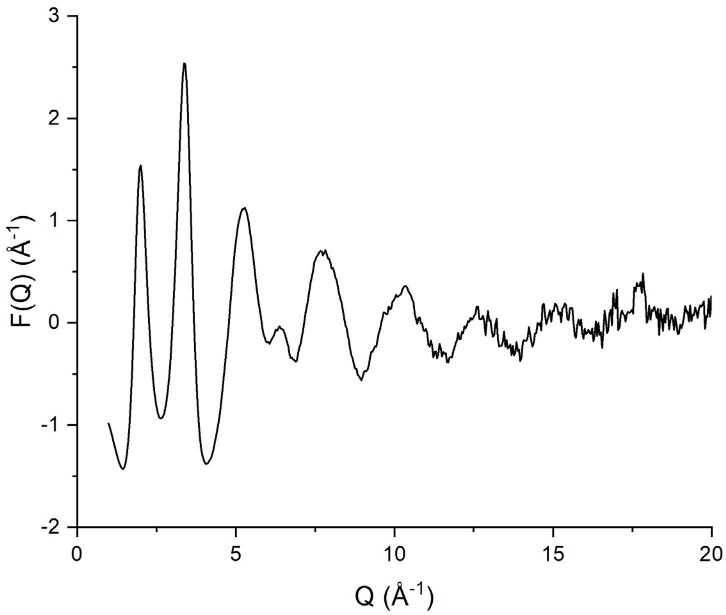

Supplementary Figure 23 | Normalized X-ray scattering function F(Q) of amorphous GeTe microparticles.

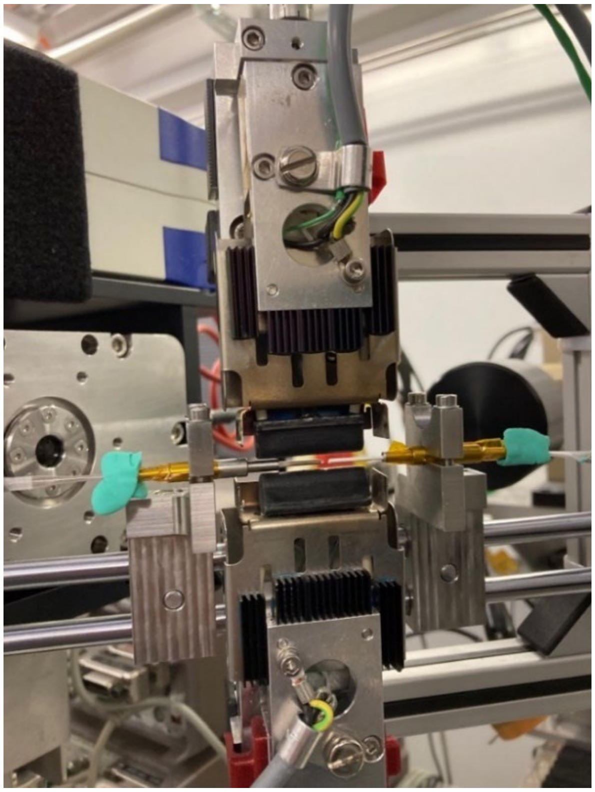

Supplementary Figure 24 | In-situ temperature X-ray absorption measuring setup. Sample capillary (left) is measured in parallel to boron nitride reference capillary (right), as placed symmetrically between two infrared heaters. Thermocouple is placed in boron nitride filled capillary and temperature is controlled using a PID controller.

Supplementary References

1. Mazzarello, R., Caravati, S., Angioletti-Uberti, S., Bernasconi, M. & Parrinello, M. Signature of Tetrahedral Ge in the Raman Spectrum of Amorphous Phase-Change Materials. *Phys Rev Lett* **104**, 085503 (2010).

2. Abou El Kheir, O., Dragoni, D. & Bernasconi, M. Density functional simulations of decomposition pathways of Ge-rich GeSbTe alloys for phase change memories. *Phys Rev Mater* **5**, 095004 (2021).

3. Guda, A. A. *et al.* Understanding X-ray absorption spectra by means of descriptors and machine learning algorithms. *npj Computational Materials 2021 7:1* **7**, 1–13 (2021).
